# Formulation and Optimization of Sustained-Release Diclofenac Microspheres for Orally Disintegrating Tablets

**DOI:** 10.1155/ijbm/5552692

**Published:** 2025-10-15

**Authors:** Meron Amdework, Fantahun Molla, Afewerk Getachew

**Affiliations:** ^1^Department of Pharmacy, Ayder Comprehensive Specialized Hospital, College of Health Sciences, Mekelle University, Mekelle, Ethiopia; ^2^Department of Pharmaceutics and Social Pharmacy, School of Pharmacy, College of Health Sciences, Addis Ababa University, Addis Ababa, Ethiopia; ^3^Department of Pharmacy, Asrat Woldeyes Health Science Campus, Debre Berhan University, Debre Berhan, Ethiopia

**Keywords:** diclofenac, microspheres, optimization, orally disintegrating tablets, sustained-release

## Abstract

**Introduction:**

Chronic musculoskeletal problems necessitate long-term symptomatic treatments. In such cases, diclofenac (D_fNa_) is frequently prescribed. However, its demand for frequent administration might result in serious dose-dependent complications. Furthermore, most patients with these illnesses are elderly and may have difficulty swallowing. Such factors can contribute to patients' noncompliance. Therefore, this study aimed to develop a sustained-release orally disintegrating D_fNa_ tablet using locally accessible excipients.

**Methods:**

D_fNa_ microspheres were prepared using the emulsion solvent evaporation technique. Several parameters, including drug-to-polymer ratio (DPR), stirring speed (SS), internal phase volume, and polyethylene glycol content, were explored for their effect on microsphere characteristics. Significant factors were then selected and further optimized to produce microspheres with desirable responses. Eventually, the optimized microspheres were compressed into orally disintegrating tablets with appropriate excipients through direct compression.

**Results:**

Preliminary studies indicated that the DPR and SS significantly influenced the response variables. Consequently, their effects on the selected response variables (entrapment efficiency [EE] and Z) were further optimized. This optimization identified optimal conditions at a DPR of 1:1.41 and SS of 905.17 rpm with a predicted EE (69.44%) and Z (175.33 μm). Confirmation tests indicated that the experimental results are in agreement with the predicted values (a percentage error below 5%). Furthermore, the three confirmation batches showed no significant difference in their characteristics, indicating remarkable reproducibility. The microspheres exhibited a non-Fickian anomalous release mechanism, best described by the Higuchi model. All the orally disintegrating tablets prepared from the microspheres met the USP specifications. However, FT1 (compressed at 10 KN) showed a release profile and kinetics similar to those of the uncompressed microspheres. Therefore, it was selected as the best formulation of D_fNa_ in this study.

**Conclusion:**

This study successfully formulated microsphere-based sustained-release orally disintegrating D_fNa_ tablets that sustained drug release for at least 12 h.

## 1. Introduction

The oral route has been the most convenient and preferred route of administration through which most drugs are administered for systemic effects [1]. Among the dosage forms administered orally, tablets are the most desirable. This is due to their ease of administration, relative stability, and convenience in manufacturing, packaging, and storage [2, 3]. However, conventional tablets need frequent dosing and are not suitable for patients with swallowing difficulty [4]. Swallowing difficulty is a common challenge prevalent in approximately 35% of the general population [5]. Similarly, frequent dosing increases the chances of missing doses and fluctuation of plasma drug levels [6]. These factors are primarily associated with non-adherence, the most common healthcare problem in treatment failure [7].

Diclofenac (D_fNa_) is an NSAID of class phenylacetic acid with anti-inflammatory, analgesic, and antipyretic effects [8]. It showed superior analgesic and anti-inflammatory efficacy compared to the other usual NSAIDs [9]. Generally, it is indicated in the treatment of musculoskeletal disorders such as rheumatoid arthritis, osteoarthritis, and other painful conditions [10]. These are common in older people for whom long-term symptomatic NSAID/D_fNa_ treatment has been used. However, D_fNa_ with a short half-life requires frequent dosing. This increases the risk of side effects, which are more pronounced when the drug is used long-term [11]. On top of this, patients with musculoskeletal disorders could also suffer from swallowing difficulties. Hence, all of these contribute to the chances of missing doses and patient non-adherence to therapy.

Swallowing difficulties posed by conventional tablets have been successfully resolved through the formulation of oral disintegrating tablets (ODTs) [4, 12, 13]. These are dosage forms intended to be placed and dispersed in the mouth (saliva) before being swallowed [14]. On the other hand, frequency of administration could be significantly reduced by designing sustained-release (SR) dosage forms in the form of single or multi-unit systems [15]. Among multiunit SR dosage forms, microspheres have gained great attention due to their small size, free-flowing nature, better drug entrapment, and good release characteristics [16, 17]. Furthermore, they reduce the risk of dose dumping, provide better drug protection, and mask undesired taste and odor [18–20]. In addition, the possibility of compressing such microspheres into ODTs will make it easy to swallow and reduce the number of doses [21, 22]. Different scholars have successfully developed SR ODT (SR-ODT) formulations, such as for metoclopramide HCl [23], amisulpride [24], domperidone [25], and D_fNa_ [26, 27]. This study aimed to evaluate and optimize parameters that affect the characteristics of SR D_fNa_ microspheres and formulate the optimized microspheres into ODTs.

## 2. Materials and Methods

### 2.1. Materials

Diclofenac sodium (Hangzhou Shine Pharma, China), ethylcellulose (Taian Ruitai Cellulose CO. Ltd., China), dichloromethane (Vav Life Sciences Pvt., Ltd., India), 96% ethanol (Desta alcohol and liquor factory, Ethiopia), Tween 80 (Nanjing Well Chemical CO. Ltd., China), mannitol (Qingdao Bright Group CO. Ltd., China), microcrystalline cellulose PH 102 (Ankjat Pulps and Bordish Lab., India), crospovidone (BASF SE, Germany), colloidal silicon dioxide (Cabot Sanmar Limited, India), magnesium stearate (Skant Health Care Ltd., India), and saccharin sodium (Tianjin Changjie Chemical CO. Ltd., China) were donated by Addis Pharmaceutical Factory S.C. (APF). Potassium dihydrogen orthophosphate (Loba Chemie Pvt., India), disodium hydrogen orthophosphate (Titan Biot Ltd, India), hydrochloric acid (Loba Chemie Pvt., India), and polyethylene glycol 400 (UNI-CHEM., India) were analytical-grade chemicals purchased from local markets in Ethiopia and were used as received.

### 2.2. Methods

#### 2.2.1. Drug Excipient Compatibility Study

The study of drug-excipient compatibility was carried out using Fourier transform infrared (FT-IR) spectroscopy (IR-Prestige-21, Shimadzu, Japan). For this purpose, the FT-IR spectra of pure D_fNa_ and its physical mixtures with ethylcellulose were obtained separately at room temperature. The scanning was performed between wave numbers 4000 to 400 cm^−1^ for each sample.

#### 2.2.2. Preparation of Microspheres

D_fNa_ microspheres were prepared using the emulsion solvent evaporation method as described by Prasanth et al. [28]. First, a specific amount of ethylcellulose was dissolved in a specific volume of the internal phase (ethanol and dichloromethane [1:1]). Then a specific amount of PEG-400 (%w/w of the EC used) was added and mixed for 10 min. Subsequently, 2 g of D_fNa_ was added and thoroughly mixed. The resulting solution was then injected drop-wise, using a 20G needle syringe, into 200 mL of aqueous solution of Tween 80 (1% w/v) under specified stirring speed (SS) for 3 h at 25°C. Finally, the microspheres obtained were filtered, washed with distilled water three times, and dried in the open air for 24 h. The compositions of the microspheres prepared for the preliminary study are presented in Table 1.

#### 2.2.3. Characterization of Diclofenac Sodium Microspheres

##### 2.2.3.1. Percentage Yield

The percentage yield of the microspheres of each batch was calculated using the actual weight of the microspheres after drying, divided by the weight of components (drug and polymer) initially used to prepare the microspheres, as shown in (1)

(1) $$\begin{array}{c} \text{Yield}\left(\%\right)=\frac{\text{Weight of dried microspheres }}{\text{Initial weight of drug and polymer }}. \end{array}$$

##### 2.2.3.2. Microsphere Size

The average microsphere size (*Z*) was analyzed using the sieve analysis method as described by Dash et al. [29]. A 20 g sample of microspheres was placed on top of standard sieves arranged in descending order (1000–180 μm). The sieves were then placed on a sieve vibrator (FRITSCH, Germany) for 10 min. Then the microspheres retained in each sieve were individually weighed, and Z was calculated according to (2)

(2) $$\begin{array}{c} Z=\frac{\sum \left(\text{Particle size of the fraction}*\text{weight fraction}\right)\;}{\sum \text{weight fractions}}. \end{array}$$

##### 2.2.3.3. Drug Entrapment Efficiency

The drug entrapment efficiency (EE) was determined using the method described by Kishore et al. [30]. Accordingly, 100 mg of microspheres was taken and crushed using a mortar and pestle. Then a 20 mg sample of the resulting powder was poured into a conical flask containing 100 mL of phosphate buffer (pH 6.8) and left for 12 h. Subsequently, the dispersion was stirred at 900 rpm for 2 h and put in a centrifuge (Sigma, Germany) at 3000 rpm for 1 h. Finally, the solution was diluted, filtered, and analyzed using a UV spectrophotometer (PG Instruments, T80, China) at 276 nm. The amount of drug in the microspheres was then calculated using (3).

(3) $$\begin{array}{c} \text{Drug entrapment efficiency}\left(\%\right)=\left(\frac{\text{Actual drug content}}{\text{Theoretical drug content }}\right)\times 100. \end{array}$$

#### 2.2.4. Calibration Curves of Diclofenac Sodium

**Figure 1:**
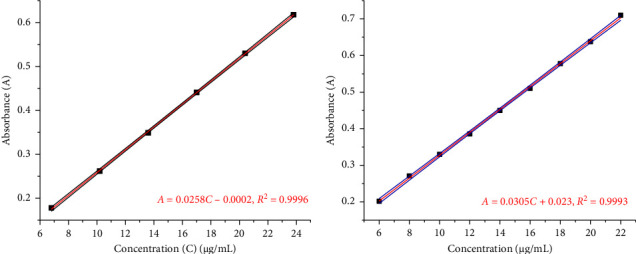
Calibration curve of reference standard diclofenac sodium in 0.1 N HCl (a) and phosphate buffer of pH 6.8 (b) with 95% confidence interval.

First, 68 g of D_fNa_ (reference) was dissolved in 0.1 N NaOH (10 mL) and diluted to 100 mL with distilled water. From this stock solution, different concentrations in the range from 6 to 24 μg/mL were prepared using a mixture of 0.1 N HCl and 5 N NaOH (900:20) as a solvent. Similar dilutions were also separately prepared using phosphate buffer (pH 6.8) [8]. Then, the absorbance was measured at a wavelength of 276 nm using a UV–visible spectrophotometer (PG Instruments, LTD, T80, China). Finally, the absorbance versus concentration graphs were plotted as depicted in Figures 1(a) and 1(b)) to obtain the calibration curves.

#### 2.2.5. In Vitro Drug Release Studies of Microspheres

The in vitro drug release of the microspheres was studied in a dissolution medium (900 mL) maintained at 37 ± 0.5°C using a USP type I (basket) apparatus rotated at 50 rpm (PHARMATEST, Germany). A mass of microspheres equivalent to 75 mg of D_fNa_ was placed in the basket, and the dissolution study was conducted in 0.1N HCl for the first 2 h and in a pH 6.8 phosphate buffer for the remaining 10 h [31]. Then 10 mL samples were removed at predetermined time intervals, filtered, diluted, and analyzed for drug content at *λ*_max_ of 276 nm. An equal volume of fresh dissolution medium kept at the same temperature was replaced in the vessel after each withdrawal to maintain the sink condition. This method was used in a similar way for the in vitro drug release study of SR-ODTs.

#### 2.2.6. Drug Release Kinetics and Mechanism

The dissolution data obtained was fitted into frequently used release kinetic models (Equations (4)–(8)) to determine the kinetics and mechanism of drug release.

(4) $$\begin{array}{c} \text{Zero}‐\text{order }Q_{t}=Q_{O}-K_{O}t, \end{array}$$

(5) $$\begin{array}{c} \text{First}‐\text{order}\ln\;Q_{t}=\ln\;Q_{o}-K_{1}t, \end{array}$$

(6) $$\begin{array}{c} \text{Higuchi square root model }M=\frac{Kt^{1}}{2}, \end{array}$$

(7) $$\begin{array}{c} \text{Hixson}–\text{Crowell }\frac{Qt^{1}}{3}=\frac{Qo^{1}}{3}-K_{HC}t, \end{array}$$

(8) $$\begin{array}{c} \text{Korsmeyer}–\text{Peppas model }\frac{Q_{t}}{Q_{0\text{⁣}}}={Kt}^{n}, \end{array}$$

where “*Q*_*t*_” (the amount of drug remaining at any time *t*), “*Q*_0⁣_” (the initial amount of drug), “*K*_0_ and *K*_1_” (the zero-order and first-order release constant, respectively), “*M*” (the amount of drug released at time t), “*K*” (the rate constant), “*K*_*HC*_” (the Hixson–Crowell rate constant), and “*n*” (a release exponent).

#### 2.2.7. Dissolution Efficiency

The dissolution efficiency was used to compare the release profiles of the formulations prepared for confirmation and was determined from the in vitro dissolution data using (9).

(9) $$\begin{array}{c} \text{DE}\left(\%\right)=\frac{\int_{t_{1}}^{t_{2}}y.dt}{Y_{100\text{⁣}}\left(t_{2\text{⁣}}-t_{1}\right)}*100, \end{array}$$

where *y* is the percentage of drug dissolved at time *t*, *Y*_100⁣_ denotes 100% dissolution, and the integral represents the area under the dissolution curve between time points *t*_1_ and *t*_2⁣_.

#### 2.2.8. Experimental Design

A preliminary study was conducted using a traditional one-factor-at-a-time technique to identify factors that significantly affect response variables such as percentage yield, EE, Z, and drug release. Then, the most significant factors and responses were selected, and further optimization was performed using the central composite design (CCD). Thus, based on the number of independent variables (*k*) and repetitions at the center point (Cp), a total of 13 experiments were carried out according to equation (2^*k*^ + 2*k* + Cp) [32]. Independent variables are studied at five levels: factorial, axial, and Cps. The axial or star points (*α*) depend on the number of variables and are calculated as *α* = 2^*k*^/4 [33]. The selected formulation variables with their limits, units, and notations are presented in Table 2.

#### 2.2.9. Formulation of SR-ODTs

The SR-ODTs were prepared by the direct compression method according to Shazly et al. [27] with some modifications. First, microspheres (equivalent to 75 mg of D_fNa_) and excipients were screened through a sieve (#60) and thoroughly mixed in a polybag for 10 min. Then magnesium stearate and aerosol were added and mixed for an additional 3 min. Finally, the powder blends were compressed in a compression machine (RIVA, MINIPRESS, Germany) using a 10 mm flat punch to a target average weight of 370% ± 5%. The compositions of these tablet formulations are provided in Table 3.

#### 2.2.10. Tablet Characterizations

##### 2.2.10.1. Tablet Hardness Test and Thickness

The hardness and thickness of 10 randomly selected tablets from each batch were determined using a hardness tester (PHARMATEST, Germany).

##### 2.2.10.2. Friability Test

Randomly selected tablets (20) were accurately weighed, placed on a friability tester (ERWEKA, Germany), and operated at 25 rpm for 4 min. The tablets were then collected, dedusted, and reweighed. Finally, friability was calculated as the percentage weight loss of the tablets.

##### 2.2.10.3. Wetting Time

The tablets were carefully placed on the surface of a doublefold soft tissue paper and placed in a Petri dish containing 10 mL of colored water. Then, the time required for the colored water to reach the upper surface of the tablet was considered as a wetting time [33].

##### 2.2.10.4. Weight Variation

Tablets (20) were individually weighed using an analytical balance (Adventurer, China). The weight variation was then calculated and presented as mean weight ± SD [8].

##### 2.2.10.5. Disintegration Time

Six tablets were placed on the disintegration tester (PHARMATEST, Germany) filled with 900 mL of distilled water kept at 37 ± 2°C^.^ Then, the time required for the complete disintegration of the tablets was considered as the disintegration time [8].

#### 2.2.11. Statistical Analysis

The results obtained were analyzed using SPSS Version 20, Microsoft Excel, and Origin 8 software (Origin Lab Corporation, USA). To compare the results, a one-way analysis of variance (ANOVA) was used. For selection and optimization, Design-Expert 7.0.0 software was used. A statistically significant difference was considered when *p* < 0.05.

## 3. Results and Discussion

### 3.1. Drug Excipient Compatibility Study

**Figure 2:**
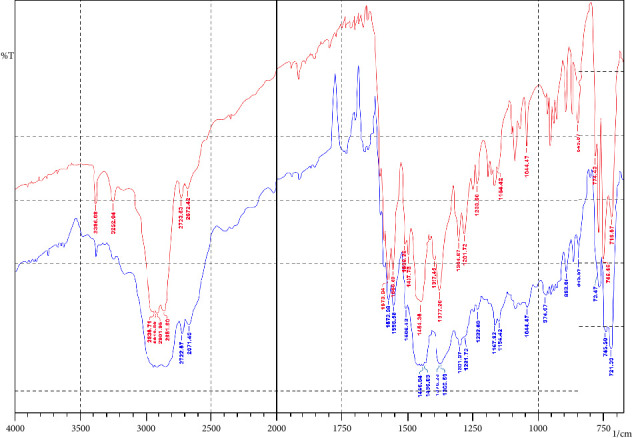
FT-IR spectrum of pure diclofenac (red) sodium and its physical mixture with ethylcellulose (blue).

The FT-IR spectrum of the physical mixture of pure D_fNa_ with EC is depicted in Figure 2. Pure D_fNa_ shows absorption bands for N-H stretching of the secondary amine group (3386 cm^−1^), C=O stretching of the carboxyl ion (1574 cm^−1^), C=C stretching (1556 cm^−1^), C-N stretching (1304 cm^−1^ and 1282 cm^−1^), and C-Cl stretching (746 cm^−1^) [34, 35]. The presence of these characteristic absorption bands of the pure D_fNa_ in the physical mixture indicated that there were no compatibility issues.

### 3.2. Preliminary Studies

In preliminary studies, the effect of drug-to-polymer ratio (DPR), PEG400 (%w/w), SS, and volume of internal phase on percentage yield, EE, Z, and cumulative drug release was studied. The results of the preliminary studies are summarized and presented in Table 4.

#### 3.2.1. Effect of the DPR

**Figure 3:**
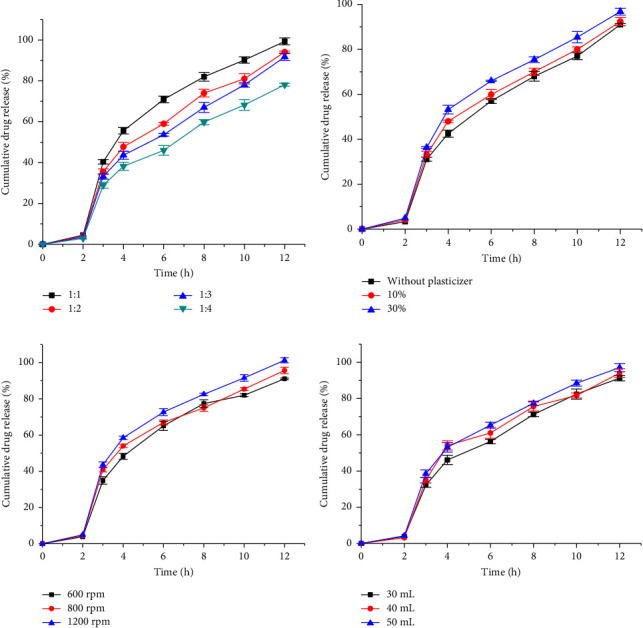
Effect of the drug-to-polymer ratio (a), PEG 400 (b), stirring speed (c), and volume of internal phase (d) on the in vitro drug release rate of the microspheres.

The DPR significantly increased the EE (*p* < 0.01), the Z (*p* < 0.001), and the percentage of microsphere yield (*p* < 0.01). This might be due to the increase in viscosity of the internal phase associated with the increase in polymer concentration. The increased viscosity prevents drug diffusion from the internal phase, enhancing the EE [36]. Similarly, it decreases the shearing capacity, resulting in large emulsion droplets; hence, bigger microspheres [37]. Furthermore, the increase in polymer concentration significantly (*p* < 0.05) retarded the drug diffusion from the microspheres (Figure 3(a)). This might be due to the increase in the thickness of the coating around the microspheres. Similar findings were reported by Shazly et al. [27] and Prasanth et al. [28].

#### 3.2.2. Effect of PEG 400

The effect of PEG on response variables, EE, Z, and percentage microsphere yield was not significant (*p* > 0.05). The results of these response variables are shown in Table 4 (MF5-7). Similarly, cumulative drug release did not show a significant difference (*p* > 0.05) as indicated in Figure 3(b). Thus, PEG was not a significant independent variable in the studied ranges and was not considered for further study.

#### 3.2.3. Effect of SS

The effect of the SS on the response variables is presented in Table 4 (MF8-10). Accordingly, Z decreased significantly as the SS increased (*p* < 0.01). This could be attributed to the high shear stress applied that breaks the droplets of the emulsion [38]. Similarly, increasing the SS decreased the percentage yield (*p* < 0.05), and EE (*p* < 0.05). This could be related to the increased extraction of the drug to the continuous phase before solidification [20]. Cumulative drug release, as depicted in Figure 3(c), increased insignificantly (*p* > 0.05) with SS, which is in line with a study done by Haznedar and Dortunç [39].

#### 3.2.4. Effect of Volume of the Internal Phase

The effect of the volume of the internal phase on the response variables of the microspheres is summarized in Table 4 (MF11-13). Consequently, Z decreased significantly (*p* < 0.05) as the volume of the internal phase increased. This is because an increase in the internal phase results in a less viscous dispersed phase by reducing the polymer concentration. Consequently, this allows the droplets to disperse to a small size while stirring [40]. On the other hand, EE and cumulative drug release did not show significant differences (*p* > 0.05). The cumulative drug release from the microspheres increased with volume, as shown in Figure 3(d). Although the increase was not significant, it might be because smaller particles possess a large surface area, which in turn increases the dissolution and drug release rates.

### 3.3. Optimization

Based on the preliminary experiments, the DPR and SS were selected as significant independent variables that require further study and optimization. Thus, the effects of these factors on the EE and Z were further studied using CCD. It is a widely employed method to explore the effects of first-order variables and their interactions while keeping the number of experiments minimal. Moreover, it employs axial points to efficiently estimate the pure quadratic effects and Cps to detect the presence of curvature in the experimental design. Therefore, a more precise estimate of the pure error and increased statistical power of the test can be obtained [41]. A total of 13 formulations, as provided by the software, were prepared and characterized (Table 5). Then, the result obtained was fed into the Design Expert Software for further analysis and prediction.

#### 3.3.1. Response Model Selection and Model Adequacy

The response model selections were made based on a comparison of several statistical parameters such as *p* values, lack-of-fit *p* values, and adjusted and predicted R-squared values [31, 42] obtained from the Design Expert Software. Therefore, the quadratic model was suggested for both the EE and Z, as provided in the model fit summary statistics (Table 6). The value of the determination coefficient (*R*^2^) indicates the goodness of fit of the model. Accordingly, the *R*^2^ values of EE (0.9485) and Z (0.9983) indicated that 94.85% and 99.83% of the variation in the response variables were attributed to independent variables, respectively.

The model adequacy test is important in understanding the relationship between the dependent and independent variables, statistical independence of errors, constant variance of errors, and normality of the error distribution based on the ANOVA result [43, 44]. Thus, as provided in Table 7, the model terms for both EE (*p* < 0.001) and Z (*p* < 0.0001) were found to be significant. The only exception was the interaction effect (AB). For that, a backward elimination was used to remove it and improve the predictability of the mode [45]. The elimination increased the *p* value for the model lack-of-fit test from 0.2055 to 0.2863 and the adequate precision ratio from 14.836 to 17.473, indicating improved predictability. For Z, all model terms were significant with an adequate precision ratio of 87.084 and an insignificant lack of fit (*p* > 0.05). Thus, the proposed models were made as adequate as possible to develop the mathematical regression equations for the EE and Z as indicated in (10) and (11), respectively.where EE (drug EE), Z (microsphere size), A (DPR), B (SS), and AB (interaction effect).


(10) $$\begin{array}{c} \text{EE}=80.36+11.99A-5.09B-4.07A^{2}-3.22B^{2}, \end{array}$$

(11) $$\begin{array}{c} Z=219.98+89.16A-13.96B-5.43AB+38.35A^{2}+8.22B^{2}, \end{array}$$

**Figure 4:**
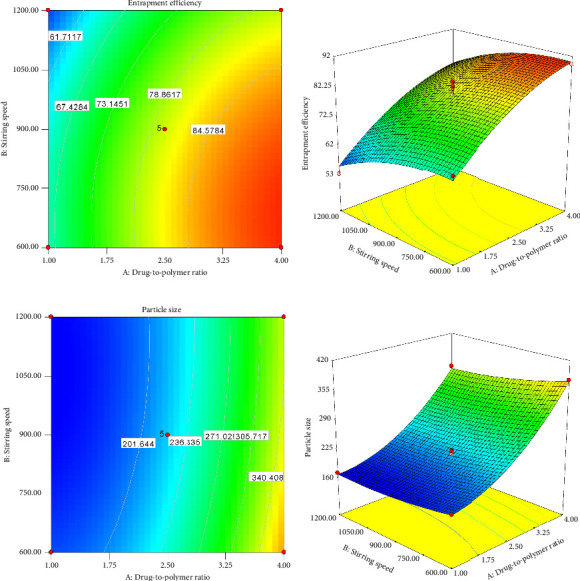
Contour and response surface plot of the drug-to-polymer ratio and stirring speed on drug entrapment efficiency (a and b) and microsphere size (c and d).

The sign and magnitude of the regression coefficients in the mathematical equation signify the relative influence of each factor on the response. The coefficients indicate the change in the response when the variable changes by a unit while other factors remain constant. Hence, a variable with a large coefficient has a significant influence on the response(s). The influence can be positive or negative depending on the sign before the coefficient of the variable [45–47]. For instance, the effect of the DPR was more pronounced on Z than on EE. Both responses were positively affected by the DPR, while the opposite was true for the SS. This phenomenon from the mathematical equations is graphically represented by 2D counter- and 3D response surface plots in Figure 4.

#### 3.3.2. Simultaneous Optimization of EE and Microsphere Size

**Figure 5:**
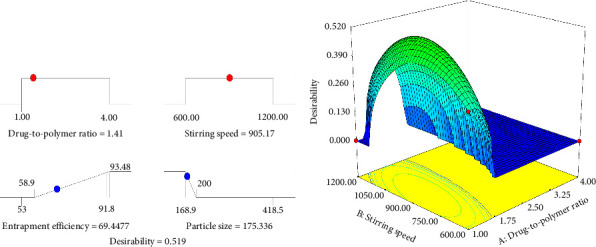
Desirability ramp for numerical optimization of variables (a) and 3D view of the overall desirability function (b).

Simultaneous optimization was carried out to maximize EE while having Z less than 200 μm. The size of the particles used in the ODT formulations should be ≤ 200 μm to avoid the stale feeling in the mouth after disintegration [25]. Therefore, both numerical and graphical optimization techniques were used with the set criteria presented in Table 8. The numerical optimization technique was used using the desirability function approach to generate an optimum setting for the formulation [42, 48]. The desirability function converts the estimated response values into individual desirability and aggregates them into a combined function. This is defined as the weighted geometric mean of all individual desirability values [49]. The total desirability in this case was 0.5, with an individual desirability of 0.3 (EE) and 0.79 (Z). The general desirability function is shown on the ramp and the 3D plot in Figures 5(a) and 5(b), respectively.

**Figure 6:**
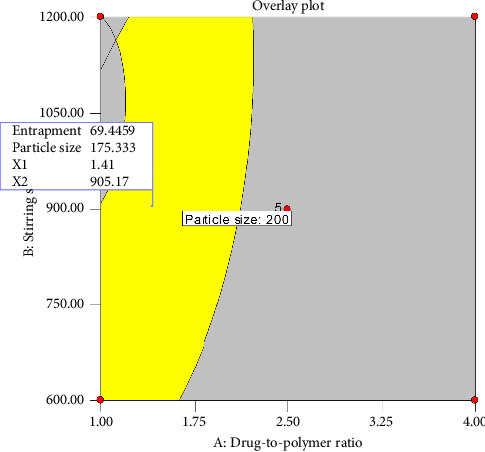
Overlay plot of drug-to-polymer ratio and stirring speed as a function of response variables entrapment efficiency and microsphere size.

Graphical optimization was carried out to visualize the optimal region for EE and Z, as depicted in Figure 6. In the figure, the yellow color indicates an area where the desired characteristics are achieved. Specifically, the point identified by the flag represents an EE of 69.44% and a Z of 175.33 μm while using a DPR of 1:1.41 and an SS of 905.17 rpm.

#### 3.3.3. Validation of Optimum Formulation

To confirm the validity of the predicted optimum results, experiments were carried out in triplicate at the optimal combination of factors (a DPR of 1:1.41 and SS of 905 rpm).  Table 9 provides the predicted values, experimental values, and percentage errors obtained at the optimum level factors. Consequently, the percentage errors were below 5%, which confirms that the experimental results are in agreement with the predicted values.

### 3.4. Evaluation of the Optimized Microspheres

#### 3.4.1. Micromeritic Evaluations

To incorporate the microspheres into a tablet dosage form, the flow properties of the microspheres are key parameters. Consequently, the results indicated that the microspheres had good flow properties based on the bulk density (0.60 ± 0.00), the tapped density (0.71 ± 0.01), the angle of repose (27.3 ± 1.15), Hausner's ratio (14.21 ± 0.11), and Carr's index (1.18 ± 0.02).

#### 3.4.2. Dissolution and Dissolution Efficiency

**Figure 7:**
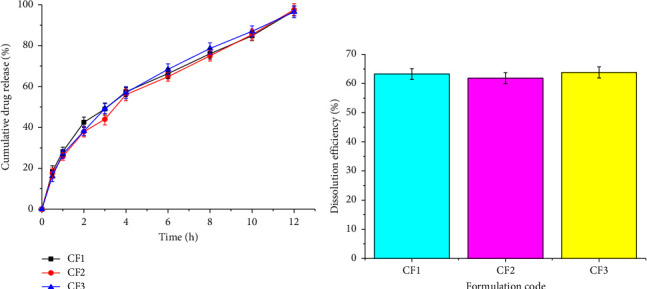
Release profiles (a) and dissolution efficiency (b) of optimized sustained-release microspheres of diclofenac sodium.

The release profile of the optimized microsphere formulation was studied for the three different batches as shown in Figure 7(a). The results indicate that there were no significant differences in the cumulative drug release profiles (*p* > 0.05). That indicates the reproducibility of the results. Similarly, the dissolution efficiency (Figure 7(b)) indicated that there was no significant difference (*p* > 0.05) in the release profile of the three batches.

#### 3.4.3. Release Kinetics of Optimized Microspheres

The release kinetics and the release mechanism of optimized microspheres are shown in Table 10. Accordingly, the Higuchi equation was the best-fit model with an *R*^2^ value of ≥ 0.9959. This might be due to the porous nature of the microspheres from the solvent evaporation method used in their preparation [50]. Similar findings were also reported in studies that used the ethylcellulose and solvent evaporation method to prepare microspheres [51, 52]. Moreover, the value of *n* was within the range of 0.45–0.89 which, according to the Korsmeyer–Peppas model, represents a non-Fickian diffusion or anomalous transport and that the drug release from microspheres was diffusion controlled through the pores of the microspheres [50, 53].

### 3.5. Precompression Evaluations

The precompression parameters of the ready powder blend for compression were evaluated as shown in Table 11. Therefore, the compressibility index, Hausner ratio, and angle of repose values indicated the free-flowing nature of the powder mixture.

### 3.6. Postcompression Evaluations

**Figure 8:**
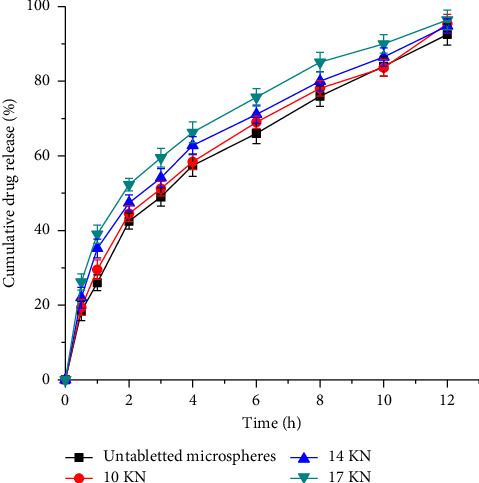
Effect of compression force on the drug release profile of sustained-release orally disintegrating diclofenac sodium tablets.

The prepared ODTs, containing a D_fNa_ microsphere, were characterized for different tablet properties. Generally, all formulations meet USP specifications for friability (< 1%), weight variation (< 5%), and disintegration time (< 1 min). The results of the tablet characteristics are presented in Table 12. To study the effect of the compression force on the microsphere release characteristics, the tablets were compressed at compression forces of 10 (FT1), 14 (FT2), and 17 KN (FT3). The cumulative drug release profiles of these compressed tablets are shown in Figure 8. Consequently, FT2 and FT3 showed 35.2% and 40.7% initial burst release, respectively, which could be due to the rupture of some microspheres due to the applied compression force. This was in line with a study done by Kasliwal et al. [23]. However, FT1, compressed at 10 KN, showed a release profile and release kinetics similar to those of the uncompressed microspheres, as shown in Table 13. Therefore, it was selected as the best SR-ODT formulation of D_fNa_ in this study.

## 4. Conclusions

This study successfully produced SR-ODT formulations from optimized D_fNa_ microspheres through the emulsion solvent evaporation method. The optimization provided optimal conditions of a DPR (1:1.41) and SS (905 rpm), where the EE and Z were 72.4% and 176.9 μm, respectively. Evaluation of the optimized microspheres showed good flow properties and sustained drug release for at least 12 h. The ODTs compressed from the microspheres complied with the specifications for an ODT. They were also able to sustain the drug release at least for 12 h, which was well described by the Higuchi drug release model. Therefore, the formulated D_fNa_ SR-ODTs might be a promising drug delivery for the treatment of musculoskeletal disorders with prolonged release, reduced dosing frequency, and improved patient compliance.

### 4.1. Limitation of the Study

The study did not include surface morphology studies (such as scanning electron microscopy), which could have contributed to a more comprehensive understanding of the microsphere's structure and its potential impact on drug release.

### 4.2. Suggestion for Further Study

The authors suggest further investigations to determine the stability of the optimized microspheres and the SR-ODTs.

## Figures and Tables

**Table 1:** The composition of diclofenac sodium microspheres prepared in the preliminary study.

Code	Drug-to-polymer ratio	PEG 400 (%w/w of EC)	Internal phase volume (mL)	Stirring speed (rpm)
MF1	1:1	15	30	600
MF2	1:2	15	30	600
MF3	1:3	15	30	600
MF4	1:4	15	30	600
MF5	1:3	0	30	600
MF6	1:3	10	30	600
MF7	1:3	30	30	600
MF8	1:3	15	30	600
MF9	1:3	15	30	800
MF10	1:3	15	30	1200
MF11	1:3	15	30	600
MF12	1:3	15	40	600
MF13	1:3	15	50	600

*Note:* Solvent = ethanol–dichloromethane (1:1), EC = ethylcellulose.

**Table 2:** Experimental levels of independent variables in the optimization study.

Variables	Limits				
	−*α*	Lower (−1)	Center (0)	Upper (+1)	+*α*
Drug-to-polymer ratio (A)	0.38	1	2.5	4	4.62
Stirring speed (B)	475.14	600	900	1200	1324.26

*Note:* −*α* and +*α* are extremely low and high star points, respectively.

**Table 3:** Compositions of sustained-release oral-disintegrating tablets of diclofenac sodium.

Form. code	Composition (mg/tablet)						
	D_fNa_	MCC	Mannitol	Crospovidone	Saccharin-Na	Mg-stearate	Aerosil
FT1	107	118	120.5	18.5	4	1	1
FT2	107	118	120.5	18.5	4	1	1
FT3	107	118	120.5	18.5	4	1	1

*Note:* FT1, FT2, and FT3 were compressed at 10, 14, and 17 KN of compression force, respectively.

**Table 4:** Effect of polyethylene glycol 400 on response variables of diclofenac sodium microspheres.

Factors		Formulation code	Response variables		
Type	Level		Percent yield (%)	Entrapment efficiency (%)	Particle size (μm)
Drug-to-polymer ratio	1:1	MF1	58.5 ± 1.76	66.8 ± 1.62	188.4 ± 1.43
	1:2	MF2	71.6 ± 1.53	75.9 ± 0.51	253.3 ± 2.67
	1:3	MF3	79.8 ± 1.30	82.3 ± 1.25	321.6 ± 1.83
	1:4	MF4	88.6 ± 1.26	89.1 ± 1.48	378.5 ± 1.04

PEG 400 (%w/w)	0	MF5	77.4 ± 2.39	78.5 ± 1.16	320.9 ± 2.28
	10	MF6	79.5 ± 1.22	81.8 ± 0.75	324.6 ± 2.15
	30	MF7	80.6 ± 2.27	82.6 ± 1.33	326.7 ± 1.33

Stirring speed (rpm)	600	MF8	79.8 ± 1.30	82.3 ± 1.25	321.6 ± 1.83
	800	MF9	77.4 ± 1.45	78.1 ± 2.32	306.6 ± 1.52
	1200	MF10	72.1 ± 2.01	70.8 ± 1.14	275.3 ± 1.45

Volume of internal phase (mL)	30	MF11	76.3 ± 1.73	83.4 ± 1.56	328.7 ± 1.28
	40	MF12	73.5 ± 0.85	82.8 ± 2.08	304.5 ± 1.63
	50	MF13	69.2 ± 1.39	76.6 ± 1.33	277.9 ± 1.54

**Table 5:** The composition and experimental results of the drug entrapment efficiency and microsphere size of the 13 formulations provided by the CCD.

Codes	Point type	Factors		Response variables	
		Drug-to-polymer ratio	Stirring speed (rpm)	Entrapment efficiency (%)	Microsphere size (μm)
FO1	Factorial	1:1 (−)	600 (−)	67.5 ± 1.04	186.5 ± 1.69
FO2	Factorial	1:4 (+)	600 (−)	89.5 ± 0.55	377.5 ± 1.80
FO3	Factorial	1:1 (−)	1200 (+)	53.0 ± 0.30	172.4 ± 2.70
FO4	Factorial	1:4 (+)	1200 (+)	76.0 ± 0.61	341.7 ± 2.56
FO5	Axial	1:0.38 (−*α*)	900 (0)	55.8 ± 1.81	168.9 ± 2.20
FO6	Axial	1:4.62 (+*α*)	900 (0)	91.8 ± 1.65	418.5 ± 2.17
FO7	Axial	1:2.5 (0)	475.74 (−*α*)	80.0 ± 1.30	255.3 ± 2.35
FO8	Axial	1:2.5 (0)	324.26 (+*α*)	71.0 ± 1.04	211.6 ± 1.75
FO9	Central	1:2.5 (0)	900 (+)	83.9 ± 0.96	215.3 ± 1.56
FO10	Central	1:2.5 (0)	900 (+)	78.4 ± 2.04	220.8 ± 1.73
FO11	Central	1:2.5 (0)	900 (+)	77.5 ± 2.25	222.7 ± 2.15
FO12	Central	1:2.5 (0)	900 (+)	79.6 ± 1.90	219.3 ± 1.05
FO13	Central	1:2.5 (0)	900 (+)	82.4 ± 1.25	221.8 ± 1.68

**Table 6:** Model fit summary statistics for drug entrapment efficiency and microsphere size.

Response	Source	*R* ^2^	Adjusted *R*^2^	Predicted *R*^2^	*p* value	Lack-of-fit *p* value	Remark
Entrapment efficiency	Linear	0.8449	0.8139	0.7412	< 0.0001	0.0705	
	2FI	0.8451	0.7934	0.5451	0.9263	0.0536	
	**Quadratic**	**0.9485**	**0.9118**	**0.7352**	**0.0211**	**0.2055**	**Suggested**
	Cubic	0.9694	0.9266	0.1808	0.2724	0.1751	Alaised

Microsphere size	Linear	0.8606	0.8328	0.7489	< 0.0001	< 0.0001	
	2FI	0.8622	0.8163	0.6739	0.7572	< 0.0001	
	**Quadratic**	**0.9983**	**0.9971**	**0.9904**	**< 0.0001**	**0.1166**	**Suggested**
	Cubic	0.9986	0.9967	0.9394	0.5850	0.0443	Alaised

*Note:* The bold values indicate the model suggested by the software, along with the corresponding statistical values for the suggested mode.

**Table 7:** Summary of ANOVA results of the response surface quadratic model for drug entrapment efficiency and size of the microspheres.

Response	Source	Sum of squares	Df	Mean square	*F*-value	*p* value	Remarks
Entrapment efficiency	Model	1523.66	5	304.73	25.81	0.0002	+
	Drug-to-polymer ratio (A)	1149.88	1	1149.88	97.38	< 0.0001	+
	Stirring speed (B)	207.35	1	207.35	17.56	0.0041	+
	AB	0.25	1	0.25	0.021	0.8884	−
	*A* ^2^	115.09	1	115.09	9.75	0.0168	+
	*B* ^2^	72.02	1	72.02	6.10	0.0429	+
	Residual	82.66	7	11.81			
	Lack of fit	53.37	3	17.79			−
	Pure error	29.29	4	7.32	2.43	0.2055	
	Cor total	1606.32	12				

Microsphere size	Model	75557.97	5	15115.60	820.60	< 0.0001	+
	Drug-to-polymer ratio (A)	63597.42	1	63597.42	3452.59	< 0.0001	+
	Stirring speed (B)	1559.64	1	1559.64	84.67	< 0.0001	+
	AB	117.72	1	117.72	6.39	0.0393	+
	*A* ^2^	10229.78	1	10229.78	555.36	< 0.0001	+
	*B* ^2^B	470.33	1	470.33	25.53	0.0015	+
	Residual	128.94	7	18.42	3.76		
	Lack of fit	95.199	3	31.73		0.1166	−
	Pure error	33.75	4	8.44			
	Cor total	75706.91	12				

*Note:* (+): significant, (−): not significant.

Abbreviation: Df, degree of freedom.

**Table 8:** Limits for factors and responses used during numerical and graphical optimization.

	Factor constraints			Response constraints			
	Low	High		Goal	Lower limit	Upper limit	Importance
A	1	4	EE	Maximize	58.9	93.48	++++
B	600	1200	Z	Minimize	168.8	200	+++++

*Note:* A (drug-to-polymer ratio), B (stirring speed), EE (drug entrapment efficiency), and Z (microsphere size).

**Table 9:** Response values of predicted and experimental value and percentage error of entrapment efficiency and microsphere size.

Response	Predicted value	Experimental value	Percentage error
EE	69.4	72.2 ± 0.47	3.87
Z	175.3	176.9 ± 2.68	0.90

*Note:* EE (drug entrapment efficiency), Z (microsphere size).

**Table 10:** Release kinetics and release mechanism of the optimized formulation.

Code	Zero-order		First-order		Higuchi		Hixson–Crowell		Korsmeyer–Peppas		
	*K*	*R* ^2^	*K*	*R* ^2^	*K*	*R* ^2^	*K*	*R* ^2^	*K*	*R* ^2^	*n*
CF1	6.99	0.9133	−0.24	0.8688	27.30	0.9959	−0.23	0.9547	3.31	0.9938	0.54
CF2	7.19	0.9376	−0.24	0.8753	27.70	0.9966	−0.23	0.9617	3.24	0.9950	0.53
CF3	7.24	0.9204	−0.23	0.9399	28.21	0.9983	−0.23	0.9839	3.23	0.9942	0.59

**Table 11:** Precompression evaluation of the powder blend for compression.

Code	Bulk density (g/mL)	Tapped density (g/mL)	Carr's index (%)	Hausner's ratio	Angle of repose (^o^)
FT1	0.66 ± 0.00	0.75 ± 0.00	12.0 ± 0.00	1.13 ± 0.00	26.6 ± 0.57
FT2	0.65 ± 0.00	0.75 ± 0.00	13.3 ± 0.34	1.15 ± 0.01	27.0 ± 1.73
FT3	0.64 ± 0.00	0.75 ± 0.01	12.9 ± 0.20	1.17 ± 0.02	27.3 ± 1.15

**Table 12:** Postcompression parameter of sustained-release orally disintegrating tablets of diclofenac sodium.

Code	Thickness (mm)	Weight (mg)	Hardness (Kgf)	Friability (%)	Wetting time (s)	Disintegration (s)
FT1	4.21 ± 0.01	374.1 ± 2.25	3.44 ± 0.37	0.61 ± 0.30	36.3 ± 2.08	20.0 ± 2.00
FT2	4.10 ± 0.03	372.2 ± 1.77	6.60 ± 0.26	0.36 ± 0.16	47.0 ± 3.60	31.6 ± 0.57
FT3	4.06 ± 0.07	369.3 ± 2.15	11.15 ± 0.66	0.26 ± 0.00	68.6 ± 3.21	56.3 ± 1.52

**Table 13:** Release kinetics and release mechanism of the selected tablet formulation.

Code	Zero-order		First-order		Higuchi		Hixson–Crowell		Korsmeyer–Peppas		
	*K*	*R* ^2^	*K*	*R* ^2^	*K*	*R* ^2^	*K*	*R* ^2^	*K*	*R* ^2^	*n*
FT1	6.8	0.8897	−0.21	0.9380	26.86	0.9937	−0.21	0.9698	3.36	0.9919	0.53

## Data Availability

The article contains all the data necessary to support the findings of this study.
